# Electroacupuncture combined with HDAC1 inhibitor suppress tumor growth via improving the recruitment of intratumor CD8^+^ T cells for triple-negative breast cancer in mice

**DOI:** 10.3389/fonc.2025.1584722

**Published:** 2025-05-22

**Authors:** Yehong Tian, Yinjie Ma, Xue Li, Gang Lu, Shixin Wang, Xiaowei Qiu, Xu Du

**Affiliations:** ^1^ College of Acupuncture and Tuina, Shaanxi University of Chinese Medicine, Xianyang, China; ^2^ Oncology Department, Wangjing Hospital of China Academy of Chinese Medical Sciences, Beijing, China; ^3^ Department of Encephalopathy Medicine, Beijing Chaoyang Integrative Medicine Rescue and First Aid Hospital, Beijing, China

**Keywords:** electroacupuncture, triple-negative breast cancer, histone deacetylase 1, CCL5, CD8+ T cells, tumor microenvironment, immune

## Abstract

Triple-negative breast cancer (TNBC) is known for its aggressive nature and poor prognosis, primarily due to limited treatment options stemming from immune evasion mechanisms. This study aimed to explore the therapeutic potential of peritumoral electroacupuncture (EA) in inhibiting tumor growth in TNBC, particularly focusing on the immune mechanisms related to CD8+ T cell recruitment and the involvement of histone deacetylase 1 (HDAC1) within the tumor microenvironment (TME). By constructing TNBC model in mice, we observed that EA not only inhibited tumor growth but also increased the presence of intratumoral CD8+ T cells and CCL5. Additionally, the expression of HDAC1 was found to down-regulate by EA. Remarkably, when EA was combined with the romidepsin (a class I HDAC inhibitor), a synergistic effect observed, leading to a greater increase in intratumoral CD8+ T cells compared to either treatment alone, resulting in a tumor inhibition rate of 60.03%. Importantly, EA did not worsen systemic inflammation, as serum levels of pro-inflammatory cytokines remained stable throughout the intervention. These findings indicate that peritumoral EA can effectively enhance anti-tumor immunity within the TME by down-regulating HDAC1. This research highlights the potential of combining non-invasive therapies like EA with pharmacological agent as a promising strategy for improving outcomes in the management of TNBC, warranting further exploration of its clinical applications.

## 1 Introduction

Triple-negative breast cancer (TNBC), defined by the absence of estrogen receptor (ER), progesterone receptor (PR) and human epidermal growth factor receptor 2 (Her2), constitutes approximately 15-20% of all breast cancer (BRCA) cases (1). This subtype of BRCA remains a formidable clinical challenge due to its aggressive biological behavior, metastatic propensity, and lack of actionable therapeutic targets (2, 3). Recent studies emphasize the significance of immune evasion in the advancement of TNBC, accentuating the promise of immunotherapy as a strategy to improve patient outcomes (4). However, fewer than 20% of TNBCs achieve durable responses to immune checkpoint inhibitors (5), largely attributed to the immunosuppressive tumor microenvironment (TME) characterized by poor CD8+ T cell infiltration and functional exhaustion (6). Emerging studies have indicated that immune cell infiltration, particularly of CD8+ T cells, plays a pivotal role in mediating anti-tumor immunity (7). The recruitment of these cytotoxic T cells to tumor sites is essential for the effective recognition and destruction of malignant cells (8).

Histone deacetylation is a crucial epigenetic mechanism that removes acetyl group of histone and plays an essential role in gene transcription. Among various cancer types, histone deacetylases (HDACs), particularly histone deacetylase 1 (HDAC1), are frequently overexpressed. This overabundance is linked to adverse outcomes and therapeutic resistance, especially in malignancies such as TNBC (9–11). Consequently, HDAC inhibitors have gained attention as critical therapeutic agents, as they enhance tumor immune antigenicity, which may slow tumor progression (12–15). Therefore, investigating the intricate relationship between immune modulation and epigenetic regulation is crucial for developing strategies to boost anti-tumor immunity (16).

Electroacupuncture (EA), a modern adaptation of traditional acupuncture from Chinese medicine, involves the insertion of needles along with the application of microcurrent to stimulate muscles and nerves. This technique has shown significant efficacy in alleviating inflammation, as evidenced by published studies that highlight its positive impact characterized by regulating inflammatory response (17, 18). Researches indicate that EA alleviates inflammation and enhances the function of immune cells and molecules in cancer patients and animal models (19–22). For instance, a randomized controlled trial showed that patients with cervical squamous cell carcinoma who received EA alongside chemotherapy exhibited significantly higher natural killer cell counts and smaller tumor volumes than those in the control group (20). Additionally, animal studies have revealed that EA, including peritumoral EA, induces a potent anti-tumor immune response (19) and effectively inhibits breast tumor growth in mouse models (19, 23–25). These findings suggest that EA holds promising potential in mitigating tumor progression and modulating immune responses, making it as a valuable therapeutic approach for breast cancer treatment. This study aims to investigate the therapeutic effects of peritumoral EA combined with HDAC1 inhibitor on tumor growth, specifically focusing on the the potential of peritumoral EA as a novel therapeutic strategy to enhance immune responses for TNBC in mice.

## 2 Materials and methods

### 2.1 Animals and cell lines

Female BALB/c mice eight to ten weeks old (Weight 20-22g) were procured from Laboratory Animal Center, Shaanxi University of Chinese Medicine (SUCM). The experimental procedures received approval of the Institutional Animal Care and Welfare Committee of SUCM (approval No. SUCMDL20230304001) and were conducted in accordance with the National Institutes of Health Guide for the Care and Use of Laboratory Animals. All mice were housed with unrestricted access to water and standard rodent chow under regulated controlled conditions with a 12h light/dark cycle and a temperature of 24 ± 2 °C. The 4T1 cell line (murine-origin TNBC cancer cells) was bought from Pricella (No.CL-0007). The 4T1 cell line transfected with luciferase (4T1-luc) was purchased from BNCC (No.241113). Cells were cultivated in RPMI-1640 (Gibco) supplemented with 10% fetal bovine serum (ExCell Bio, FSP500) and 1% penicillin-streptomycin liquid (only used to 4T1, not 4T1-luc) at 37 °C and 5% CO_2_ in an incubator.

### 2.2 Tumor model establishment

4T1-luc cells were mainly used for in bioluminescence imaging and 4T1 cells were used for flow cytometry (FCM), immunofluorescence and cytokines assay. Cells in the logarithmic phase were suspended in phosphate-buffered saline (PBS). An orthotopic breast tumor model was established by subcutaneously injecting 100 μL of cell suspension (0.5*10^6^ cells) into the 4th left inguinal mammary gland fat pad. The control group mice were injected with 100 μL of PBS in the same area. The perpendicular diameters (width, W; length, L) of the tumors were measured by a Vernier caliper. Tumor volume (V) was calculated according to the following formula: V = (L ×W^2^)/2. Mice implanted with 4T1 (4T1-luc) cells were randomly distributed to corresponding groups when the tumor volume reached at 50 mm^3^ (26).

### 2.3 Peritumoral EA intervention

The mice received peritumoral EA intervention with 2% isoflurane (RWD, No.R5102216) anesthesia. The sterilized acupuncture needles (0.18 × 13 mm, Zhongyan Taihe, China) was conducted at a distance of 2 mm from the tumor with four needles equidistantly placed at 90° intervals to encircle the tumor (total 360°). Then, the needles were inserted into the tumor’s base to a depth of approximately 3 to 5 mm. The positive and negative electrodes of the EA device was connected to two needles apart at 180° intervals, respectively. Thereby, the electric current was designed to traverse the tumor region during stimulation. The stimulation of EA device (Han’s Point-Nerve Stimulator Instrument, No.LH202H) was administered at an intensity of 0.1 mA and a frequency of 2/30 Hz for a duration of 10 minutes. The peritumoral EA intervention performed every other day. Mice in the control and the model groups received only isoflurane anesthesia, while did not receive the peritumoral EA intervention. **Supplementary Figure 1** shows a visual representation of the EA intervention applied to tumor in mice.

### 2.4 Administration of HDAC1 inhibitor

To determine if peritumoral EA combined with the class I HDAC inhibitor Romidepsin (HY-15149, MCE) enhances CD8+ T cell recruitment and tumor inhibition, Romidepsin was administered intraperitoneally at a single dose of 2 mg/kg dissolved in sterile saline every three days (12). The mice in the vehicle group were intraperitoneally injected with sterile saline.

### 2.5 Bioluminescence imaging

Tumor-bearing mice received an intraperitoneal injection of 200 μL of D luciferin potassium (MCE, HY-12591B) reconstituted in DPBS at a dosage of 150 mg/kg. The mice were subjected to 3% isoflurane anesthesia within an induction chamber connected to a V-1 bench anesthesia machine (VetEquip, #1806). Following a ten-minute interval post-injection of D luciferin potassium, the mice were transferred to an *in vivo* imaging system (PerkinElmer) for image acquisition. The imaging exposure duration was established at 1 second. During the imaging process, the mice were maintained under 2% isoflurane anesthesia through a mask on the V-1 anesthesia machine. Prior to exporting the images, the luminescence intensity scale was standardized across all images. Tumor volume was assessed by quantifying the bioluminescence signal, specifically the total photon flux, utilizing the ‘region of interest’ (ROI) measurement tools available in the Living Image software (version 4.8, Revvity).

### 2.6 FCM

Peripheral blood was collected from mice via orbital sinus bleeding and mixed into anticoagulant-coated EP tubes, then left at room temperature.The mouse spleens were removed, rinsed with pre-cooled PBS, and placed on a 70 μm cell strainer. The spleens were then ground using a sterile syringe plunger while adding the pre-cooled PBS. The resulting cell suspension was collected and centrifuged at 3500 g for 5 minutes at 4°C. Tumor tissues excised from tumor-bearing mice were processed to prepare cell suspensions in accordance with the guidelines provided by the manufacturer (130-096-730, Miltenyi Biotec). The anticoagulated whole blood, spleen, or tumor single-cell suspensions (100 μL) were incubated with 2 μL CD3-FITC antibody (200204, BioLegend) and 2 μL CD8-APC antibody (100712, BioLegend) at room temperature for 30 minutes. After incubation, 500 μL of red blood cell lysis buffer was added, and the mixture was incubated at room temperature for 15 minutes. The cells were then washed twice with 2 mL of PBS by centrifugation at 1000 rpm for 5 minutes. Finally, the cell pellet was resuspended in 200 μL of PBS for flow cytometric analysis (ACEA, NovoExpress 1.5.6). Subsequent analysis of the data was conducted utilizing FlowJo software (Tree Star).

### 2.7 Immunofluorescence

After intraperitoneal injection of 20% urethane to euthanize the mice, tumor tissues were collected and fixed in 4% paraformaldehyde, then dehydrated in 15% and 30% sucrose solutions correspondingly, embedded in OCT, sectioned at 10 μm thickness on a cryostat (NX50, Thermo, Germany), and stored at -20°C. For immunofluorescence staining, sections were washed with PBS, permeabilized with 0.3% Triton-100, blocked with 5% BSA, incubated with primary antibodies with appropriate dilution concentration (room temperature for 1 hour, 4°C overnight), washed with PBS, then incubated with fluorescently labeled secondary antibodies (37°C for 1 hour) in the dark, stained with DAPI for nuclei, and mounted with anti fluorescence quenching sealing agent (L, AC28L532). Information about the primary antibodies are listed in **Table 1**. Sections were observed on a fluorescence microscope (DM4B, Leica, Germany), with channels for Alexa 594, Alexa 488, and DAPI (AR1176, Boster) selected. After adjusting the light intensity, six random fields were selected under ×400 magnification. The number of CD8+ T cells and TH+ nerve fibers was counted. The fluorescence intensity of HDAC1 was analyzed using Image J.

**Table 1:** Information on the primary antibodies used in this study.

Antibody	Host Species	Antibody Code	Company	Dilution
Anti-CD8 alpha	Rabbit	ab217344	Abcam, England	1:500
Anti-HDAC1	Rabbit	ab109411	Abcam, England	1:200
CCL5	Mouse	sc-365826	Santa Cruz, USA	1:300
CD8A	Mouse	sc-70802	Santa Cruz, USA	1:300

### 2.8 Cytokines assay

Blood samples were collected and allowed to coagulate at ambient temperature for two hours. Subsequently, these samples underwent centrifugation for 15 minutes at 2500 rpm, facilitating the separation of the serum supernatant. To measure the concentrations of key pro-inflammatory cytokines, including IL-6 (EK0411, BOSTOR), TNFα (EK0527, BOSTOR), TGFβ (EK0515, BOSTOR), and CCL5 (EK0495, BOSTOR), a Mouse Pro-inflammatory V-Plex Tissue Culture Kit (MesoScale Discovery, Rockville, MD) was employed, adhering to the manufacturer’s protocols. For the cytokine assay, both the standard solutions and the diluted serum samples were incubated in multi-spot plates for two hours at room temperature. Following the incubation, the plates were subjected to an additional two-hour incubation with detection antibodies. After the addition of read buffer T, the plates were evaluated utilizing a MESO QUICKPLEX SQ120 instrument to quantify the electrochemiluminescence signals. In parallel, frozen tumor tissue was retrieved, and a 100 mg portion was cut from the center and placed into an EP tube. Protein lysis buffer was added at six times the volume of the tissue weight (6 μL/mg). The tissue homogenizer was cooled to 4°C, the EP tube was placed into the homogenizer, then it was homogenized at 6500 rpm for 15 seconds, repeated six times with 20-second intervals. Following homogenization, the EP tube was removed and placed on ice for an additional 30 minutes to facilitate lysis. The sample was then subjected to low-temperature high-speed centrifugation at 12000 rpm for 20 minutes at 4°C. The supernatant was transferred to a new EP tube for the detection of CCL5 (EK0495, BOSTOR) as described above, ensuring accurate measurement of cytokine concentrations by referencing standard calibration points.

### 2.9 Statistical analysis of experimental data

The statistical analyses were conducted using the R software version 4.2.1. Depending on the data format, appropriate statistical methodologies were selected, utilizing the stats package [4.2.1] and car package [3.1-0] for analysis, while the ggplot2 package [3.3.4] facilitated data visualization. All data are expressed as means ± standard error of the mean (SEM). For comparisons between two groups, statistical assessments were conducted using the T-test, followed by either Welch’s t-test or the Wilcoxon rank sum test as necessary. When comparing variables across more than two groups, one-way ANOVA was employed, followed by Welch’s one-way ANOVA, Tukey HSD or the Kruskal-Wallis test as deemed appropriate. Furthermore, the Games-Howell test was utilized for *post hoc* analysis subsequent to ANOVA in order to evaluate the differences among the group means. A *p*-value of less than 0.05 was regarded as indicative of statistical significance.

### 2.10 Bioinformatics analysis

The Xiantao Academic platform (https://www.xiantao.love/) (27) was employed for pan-cancer differential expression analysis of HDAC1, utilizing data from 33 cancer types sourced from the The Cancer Genome Atlas (TCGA, https://portal.gdc.cancer.gov/). An independent sample differential analysis for BRCA was subsequently performed using the TCGA and TCGA_GTEx datasets, respectively. To confirm and substantiate the above results, a paired differential analysis was executed on the BRCA dataset from TCGA. The RNA sequencing data along with corresponding clinical data were extracted from the TCGA database in level 3 HTSeqfragments per kilobase per million (FPKM) format, which was subsequently converted into transcripts per million reads (TPM) format and analyzed after applying log_2_ transformation. For statistical evaluation and data visualization, the ggplot2 package [3.3.4], along with the stats package [4.2.1] and car package [3.1-0], were employed. The analysis of two data groups involved the implementation of the Shapiro-Wilk normality test, the Kruskal-Wallis test, and a multiple hypothesis testing approach (Dunn’s test) that incorporated Bonferroni adjustment for significance levels. Additionally, to elucidate the expression profile of HDAC in TNBC, the UALCAN database (https://ualcan.path.uab.edu/) was utilized to explore the correlation between HDAC1 gene expression and various histological subtypes of BRCA. Furthermore, to clarify the clinical relevance of HDAC1 in both diagnosis and prognosis, we assessed the diagnostic capability of HDAC1 for BRCA utilizing the receiver operating characteristic (ROC) curve on the Xiantao Academic website. The pROC program [1.18.0] facilitated the ROC analysis, and the ggplot2 package [3.3.4] was employed for visual representation of the results. Ultimately, we examined the influence of HDAC1 on overall survival (OS) using online Kaplan-Meier (KM) Plotter (http://kmplot.com/analysis/) (28). A *p*-value of less than 0.05 was considered statistically significant.

## 3 Results

### 3.1 EA suppressed tumor growth in mice implanted with orthotopic mammary tumors

**Figure 1:**
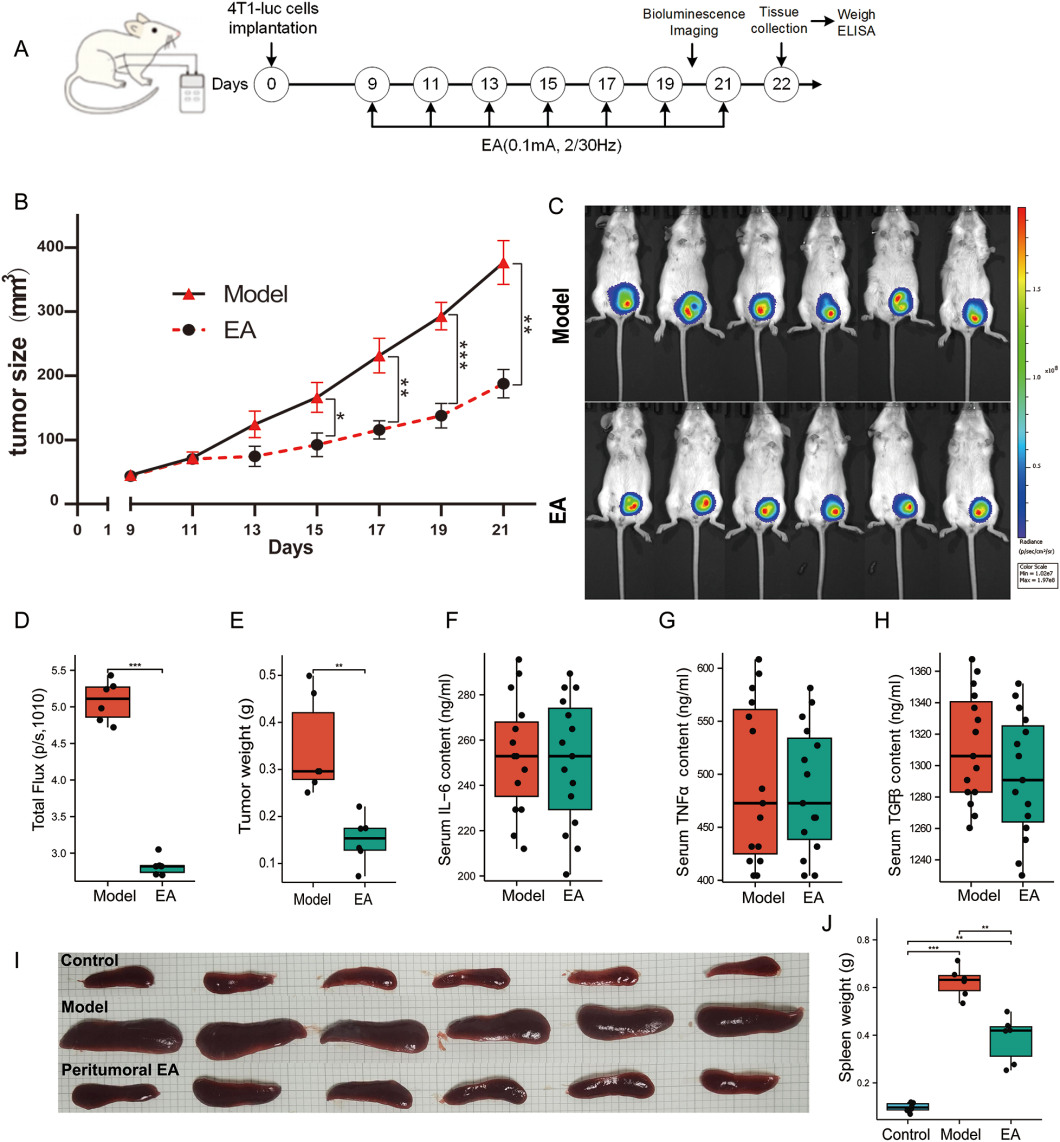
EA restrained tumor growth in mice with orthotopic mammary tumors. **(A)** The experimental timeline illustrated schematically, indicating that peritumoral EA intervention was administered every other day, commencing on the 9th day post-implantation of 4T1-luc cells and continuing until the 21st day. **(B)** The tumor growth curves for both groups were recorded bi-daily and measured using Vernier calipers (n = 6, Welch’s t’ test). **(C)** Bioluminescence imaging between the two groups on the 20th day. **(D)** The total fluorescence flux of tumors between the two groups (n = 6, Welch’s t’ test). **(E)** Tumor weight between the two groups on the 21st day(n = 6, Welch’s t’ test). **(F–H)** Serum levels of the cytokines IL-6 **(F)**, TNFα **(G)**, and TGFβ **(H)** in both groups on the 22nd day using ELISA (n = 6, with three independent measurements, Student’s t-test). **(I)** Photographs of spleens harvested from the three groups of mice on the 22nd day, with a scale bar indicating 1 cm. **(J)** Spleen weights for the three groups of mice on the 22nd day were compared (n = 6, Welch one-way ANOVA followed by Games-Howell *post hoc* test). All data are expressed as means ± SEM, with statistical significance indicated as * *p* < 0.05, ** *p* < 0.01, *** *p* < 0.001.

All mice exhibited detectable tumors (50mm^3^) within 8 days following the inoculation of 4T1-luc cells, which have been confirmed as TNBC lacking ER, PR and Her2. As illustrated in **Figure 1A**, peritumoral EA intervention was administered once every other day commencing on the ninth day post-implantation. The parameters and duration of EA intervention was derived from our preliminary observation (24, 29). Every two days, we calculated the tumor volume from the perpendicular diameters including width and length using the formula provided in section 2.2, and then we used this volume to construct the tumor growth curve. As shown in the **Figure 1B**, a significant decrease in tumor volume was noted from the 15th day (*p* < 0.05) to the 21st day (p < 0.01) in the EA group as compared to the model group. Given that 4T1-luc cells were stably transfected with luciferase, the tumor burden was tracked through bioluminescence analysis with an IVIS imaging system. Bioluminescence imaging was performed *in vivo* on the 20th day (**Figure 1C**). Following EA treatment, both the fluorescence area and intensity of the localized tumors were markedly lower than those observed in the model group (*p* < 0.001, **Figures 1C, D**). On the 22nd day, tumors were excised for weight measurement, which also reflected a significant reduction in solid tumor weight following peritumoral EA intervention (*p* < 0.01, **Figure 1E**). To verify the safety of EA in tumor treatment, we used ELISA to detect circulating inflammation cytokines IL-6, TNFα and TGFβ, which significantly contribute to tumor progression (30, 31). The results showed that the level of cytokines IL-6, TNFα and TGFβ following the EA intervention were not significantly increased compared to the model group (*p* > 0.05, **Figures 1F–H**). In addition, EA markedly alleviated splenomegaly (*p* < 0.01, **Figures 1I, J**), which was one of the common consequences in mice bearing orthotopic breast tumors (32). Overall, these results indicated that peritumoral EA effectively inhibits tumor growth without increasing the levels of circulating pro-inflammatory factors for TNBC in mice.

### 3.2 EA elevated the proportion of CD8+ T cells and promoted intratumoral CCL5 level

**Figure 2:**
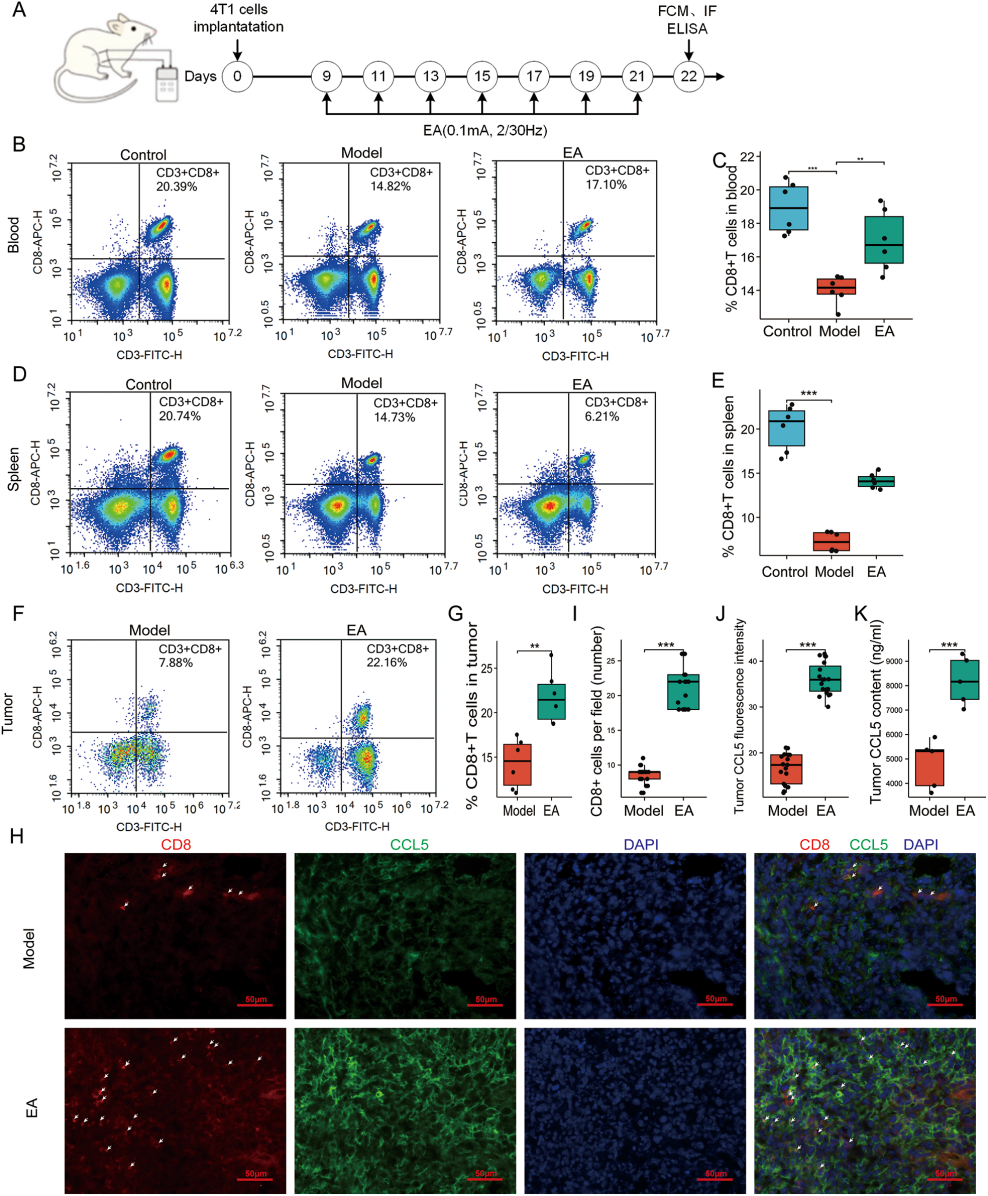
EA promoted the recruitment of CD8+ T cells and increased intratumoral CCL5 production. **(A)** Schematic representation of the experimental timeline. **(B)** Representative flow cytometry (FCM) analysis of CD8+ T cells in the blood. **(C)** Proportions of CD8+ T cells (CD3e+ CD8a+) in the blood of mice in the 3 groups (n = 6, one-way ANOVA followed by Tukey HSD). **(D)** Representative FCM analysis of CD8+ T cells in the spleen. **(E)** Proportions of CD8+ T cells in the spleen of mice in the 3 groups (n = 6, Kruskal-Wallis Test). **(F)** Representative FCM analysis of CD8+ T cells in the tumor. **(G)** Proportions of CD8+ T cells in the tumor of mice in the 2 groups (n = 6, Student’s t-test). **(H)** Representative immunofluorescence images of CD8 (red, arrowhead) and CCL5 (green) in the tumor tissue in the 2 groups. Scale bar, 50 μm. **(I)** Proportions of CD8+ T cells in the tumor in the 2 groups detected by immunofluorescence (n = 6, Student’s t-test). **(J)** Relative expression of CCL5 in the tumor of mice in the 2 groups detected by immunofluorescence (n = 3, Student’s t-test). **(K)** Tumorous levels of the CCL5 in the 2 groups detected by ELISA (n = 6, Student’s t-test). All data are presented as the means ± SEM, **p < 0.01, ***p < 0.001.

Knowing that CD8+ T cell play critical roles in antitumor immunity (33, 34). Elevated levels of CD8+ T cells are associated with improved outcome in patients with BRCA (35). In this study, we evaluated CD8+ T cells in the blood, spleen and tumor on the 14th day following the EA intervention (**Figure 2A**). The FCM findings showed that the proportions of CD8+ T cells in the blood and spleen were markedly diminished on the 22nd day post-implantation (*p* < 0.001, **Figures 2B–E**), implying that host antitumor immunity was compromised in breast tumor mice. The EA intervention effectively mitigated the decline of CD8+ T cells in the blood (*p* < 0.01, **Figures 2B, C**). Additionally, while the EA reduced the decline of CD8+ T cells in the spleen (*p* > 0.05, **Figures 2D, E**), no significant distinction was observed between the model group and the EA group. In local tumors, FCM analysis revealed that EA intervention significantly increased the proportion of CD8+ T cells (*p* < 0.01 **Figures 2F, G**). CCL5 is the most relevant chemokine in terms of the spatial distribution of intratumoral CD8+ T cells in pancreatic ductal adenocarcinoma (36), it is not elucidated in TNBC. Therefore, we conducted immunofluorescence analysis of co-staining with CD8+ T and CCL5. The results showed that CD8+ T cells in the tumor was significantly increased after EA (*p* < 0.001, **Figures 2H, I**), which was consistent with the analysis of FCM. Correspondingly, EA significantly increased the expression of CCL5 in the tumor tissue *p* < 0.001, **Figures 2H, J**). Meanwhile, The CCL5 in local tumor was also detected by ELISA, and the result was consistent with the above result of immunofluorescence (*p* < 0.001, **Figure 2K**). In addition, ELISA was used to detect serum CCL5, which showed that EA did not affect the circulating CCL5 level (*p* > 0.05, **Supplementary Figure 2**). Taken together, these data indicated that EA improved the proportion of CD8+ T cells and and promoted intratumoral CCL5 level.

### 3.3 HDAC1 is related with the prognosis of TNBC, while EA diminished the expression of HDAC1

**Figure 3:**
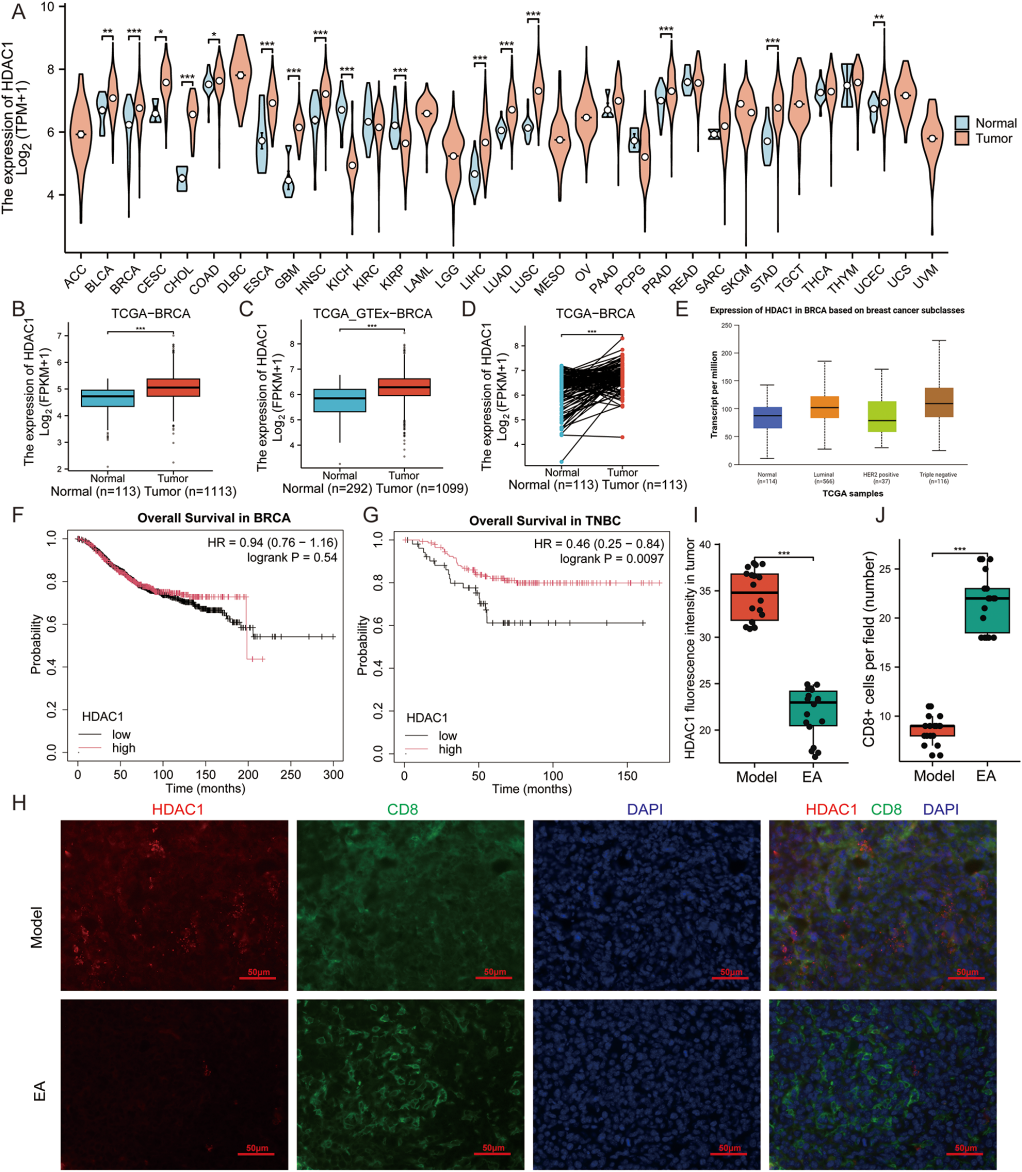
HDAC1 is associated with the prognosis of TNBC and EA diminished the expression of HDAC1. **(A)** HDAC1 expression is elevated in 16 tumor tissues including BRCA in the The Cancer Genome Atlas (TCGA). **(B, C)** HDAC1 mRNA expression levels in the TCGA-BRCA **(B)** and TCGA_GTEx-BRCA **(C)**. **(D)** HDAC1 mRNA expression levels in BRCA patients and matched adjacent normal samples in the TCGA-BRCA. **(E)** Associations between HDAC1 expression and different histological subtypes in BRCA analyzed by the UALCAN database. **(F, G)** Kaplan-Meier (KM) plots showing HDAC1’s influence on undifferentiated overall survival (OS) in BRCA patients **(F)**, while poorer OS in TNBC patients **(G)** from the KM Plotter website. **(H)** Representative immunofluorescence images of HDAC1 (red) and CD8 (green) in the tumor in the 2 groups. Scale bar, 50 μm. **(I)** Relative expression of HDAC1 in the tumor of mice in the 2 groups detected by immunofluorescence (n = 3, Wilcoxon rank sum test). **(J)** Proportions of CD8+ T cells in the tumor of mice in the 2 groups detected by immunofluorescence (n = 3, Wilcoxon rank sum test). All data are presented as the means ± SEM, *p < 0.05, **p < 0.01, ***p < 0.001.

Histone deacetylase 1 (HDAC1) is a crucial enzyme in the domain of epigenetic regulation, functioning in conjunction with histone acetyltransferases (HAT) to maintain the balance of histone acetylation (37). Its significance extends beyond chromatin remodeling, as recent research has highlighted HDAC1’s crucial role in antiviral immunity, facilitating the body’s defense mechanisms against viral pathogens through the modulation of immune response (38). Notably, HDAC1 has emerged as a key prognostic marker in glioma (39) and is increasingly scrutinized for its potential role in immune evasion, particularly in TNBC. In our study, comprehensive analyses utilizing mRNA sequencing data from The Cancer Genome Atlas (TCGA) have demonstrated that HDAC1 expression is markedly elevated in 16 different tumor types including BRCA compared to normal tissues (**Figure 3A**). Specifically, HDAC1 was found to be highly expressed in BRCA tissues relative to adjacent normal tissue (*p* < 0.001, **Figures 3B–D**). Further investigations utilizing the UALCAN database revealed that HDAC1 expression was particularly elevated in TNBC, suggesting a potential link between HDAC1 and aggressive cancer phenotypes (**Figure 3E**). Additionally, the diagnostic and prognostic implications of HDAC1 in BRCA were assessed, revealing an area under the ROC curve of 0.714 (**Supplementary Figure 3**), indicating a relatively high accuracy in diagnosing BRCA. Interestingly, elevated HDAC1 expression did not correlate with poorer OS in general BRCA patients (*p* = 0.54, **Figure 3F**). However, it was associated with worse OS in patients diagnosed with TNBC (*p* = 0.0097, **Figure 3G**). Furthermore, our *in vivo* study indicated that peritumoral EA significantly diminished HDAC1 levels for TNBC model in mice (p < 0.001, **Figure 3I**), correlating with an increase in CD8+ T cell infiltration in the TME (*p* < 0.001, **Figures 3H, J**). These findings underscore the essential role of HDAC1 in BRCA, particularly in the TNBC, and highlight EA as a promising therapeutic strategy.

### 3.4 HDAC1 inhibitor improved EA to recruit intratumoral CD8+ T cells and CCL5

**Figure 4:**
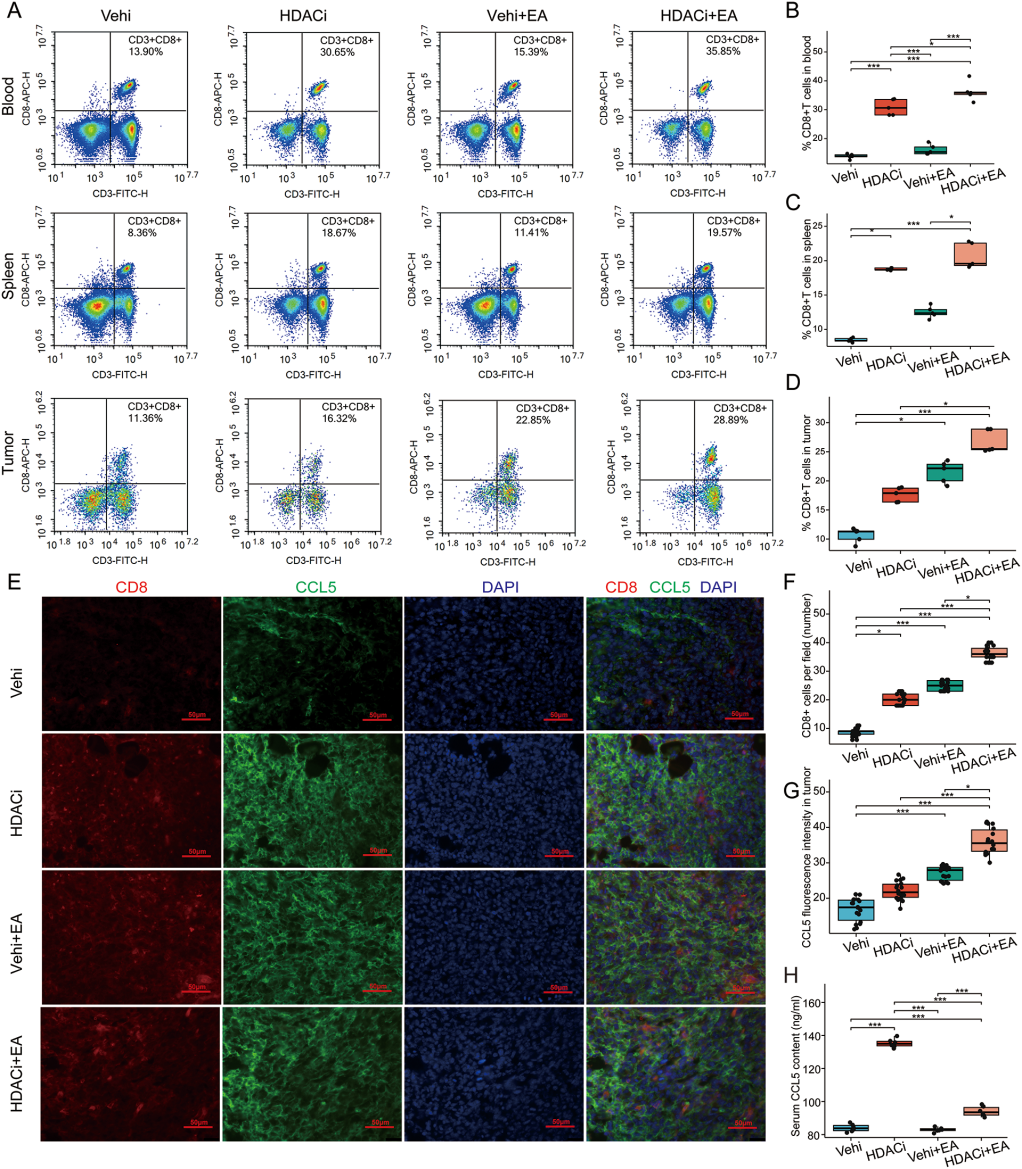
HDAC1 inhibitor enhanced EA to augment intratumoral CD8+ T cells and CCL5. **(A)** Representative FCM analysis of CD8+T cells in the blood, spleen and tumor. **(B–D)** Proportions of CD8+T cells in the blood (B, n = 5, one-way ANOVA followed by Tukey HSD), spleen (C, n = 5, Kruskal-Wallis Test followed by Dunn’s test) and tumor (D, n = 5, Kruskal-Wallis Test followed by Dunn’s test) of mice in the 4 groups. **(E)** Representative immunofluorescence images of CD8 (red) and CCL5 (green) in the tumor in the 4 groups. Scale bar, 50 μm. **(F)** Proportions of CD8+ T cells in the tumor of mice in the 4 groups detected by immunofluorescence (n = 3, Kruskal-Wallis Test followed by Dunn’s test). **(G)** Relative expression of CCL5 in the tumor of mice in the 4 groups detected by immunofluorescence (n = 3, Kruskal-Wallis Test followed by Dunn’s test). **(H)** Tumorous levels of the CCL5 in the 4 groups detected by ELISA (n = 5, Welch one-way ANOVA test followed by Games-Howell). All data are presented as the means ± SEM, *p < 0.05, ***p < 0.001.

Recent studies have highlighted the role of HDAC1 in maintaining an immune-suppressive TME (15). Specifically, it has been observed that the use of the romidepsin (a class I HDAC inhibitor) leads to a significant increase in CCL5 levels, thereby enhancing the immune response in various cancers, including gynecologic cancers and melanoma (13–40). Our previous findings indicated that EA specifically down-regulated HDAC1 expression, prompting us to investigate whether this downr-egulation contributes to immune escape for TNBC in murine models. FCM analysis revealed that romidepsin significantly elevated the levels of CD8+ T cells in both blood and spleen, while it did not markedly affect intratumoral CD8+ T cells (**Figures 4A–D**). Notably, when combining the HDAC1 inhibitor with EA intervention, there was a pronounced increase in intratumoral CD8+ T cell levels (**Figures 4A, D**). Immunofluorescence confirmed that the HDAC1 inhibitor enhanced EA’s ability to recruit intratumoral CD8+ T cells (**Figures 4E, F**). Furthermore, both immunofluorescence and ELISA demonstrated that the combination of the HDAC1 inhibitor and EA significantly augmented intratumoral CCL5 levels (**Figures 4E, G**, **Supplementary Figure 4**). EA alone did not significantly impact serum CCL5 levels, while the application of the HDAC inhibitor resulted in a substantial increase (**Figure 4H**). Notably, when EA was combined with the HDAC inhibitor, there was a reduction in serum CCL5 levels compared to the use of the HDAC inhibitor alone (**Figure 4H**). These results collectively suggest that inhibiting HDAC1 enhances EA’s efficacy in recruiting intratumoral CD8+ T cells by upregulating CCL5 production in the TME.

### 3.5 EA-mediated alleviation of tumor growth was enhanced by inhibiting HDAC1

**Figure 5:**
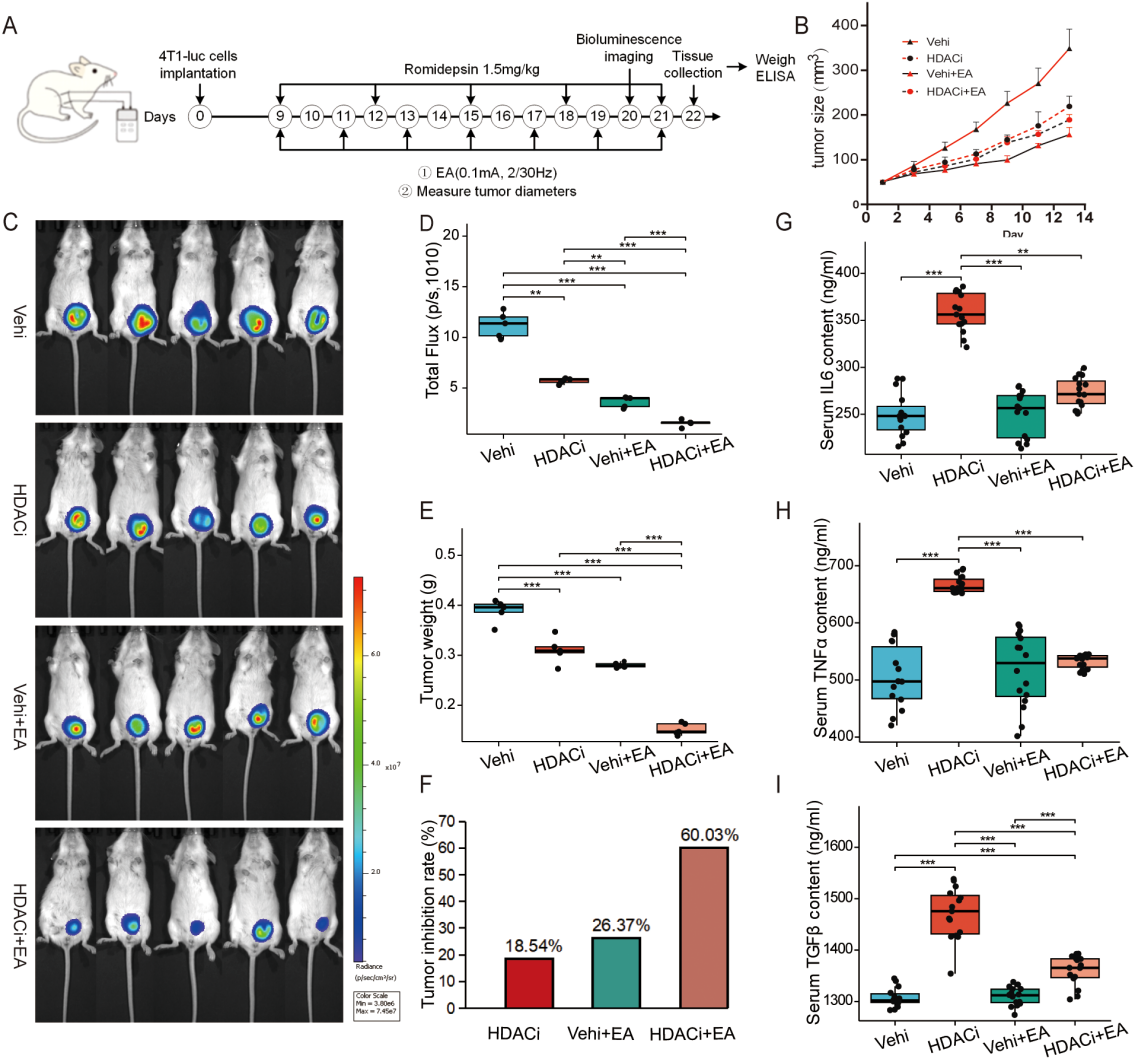
Inhibiting HDAC1 enhanced the suppressive effect of EA on tumor growth. **(A)** Schematic representation of the experimental timeline. **(B)** Tumor growth curves in the 4 groups every other day measured by Vernier calipers (n = 5, Welch one-way ANOVA test followed by Games-Howell). **(C)** Bioluminescence imaging on the 20th day in the 4 groups. **(D)** Total fluorescence flux of the tumor between the 4 groups (n = 5, Welch one-way ANOVA test followed by Games-Howell). **(E)** Comparison of tumor weight between the 4 groups on the 22nd day (n = 5, One-way ANOVA followed by Tukey HSD). **(F)** Tumor inhibition rate in the 3 group. **(G–I)** Serum levels of the cytokines IL-6 (G, n=5, measurements were repeated three times, One-way ANOVA followed by Tukey HSD), TNFα (H, n=5, measurements were repeated three times, Kruskal-Wallis Test followed by Dunn’s test) and TGFβ (I, n=5, measurements were repeated three times, Welch one-way ANOVA test followed by Games-Howell) in the 4 groups detected by ELISA. All data are presented as the means ± SEM, **p < 0.01, ***p < 0.001.

To explore the therapeutic potential of HDAC1 inhibitor in enhancing EA-mediated tumor suppression, we meticulously assessed tumor growth curve, bioluminescence imaging, tumor weight, and systemic pro-tumorigenic cytokine levels in the TNBC transplant model (**Figure 5A**). Tumor growth curves revealed that both EA and HDAC1 inhibitor retarded tumor progression compared to the vehi group (**Figure 5B**). Notably, the combination of EA with the HDAC1 inhibitor precipitated the most pronounced tumor shrinkage (**Figure 5B**). Subsequently, tumor bioluminescence imaging *in vivo* corroborated this observation. Tumors in the HDACi + EA group displayed significantly diminished bioluminescent signals than those in the HDACi group and EA group (**Figures 5C, D**). After the experimental intervention, the tumors were collected and weighed. The tumor weights in the HDACi + EA group were remarkably reduced compared to those in the HDACi groups (**Figure 5E**). The tumor inhibition rates were 18.54% for HDACi group and 26.37% for EA group. The synergistic application of EA and HDACi elevated this rate to 60.03% (**Figure 5F**). To evaluate potential off-target effects, we quantified serum levels of IL-6, TNFα, and TGFβ, the key cytokines associated with tumor progression. Following the intervention of HDACi, these cytokines were significantly elevated (*p* < 0.001). However, no notable increase was observed in the HDACi + EA group (**Figures 5G–I**). These findings confirm that the inhibition of HDAC1 amplifies the antitumor efficacy of EA without inciting systemic inflammation.

## 4 Discussion

With the high rates of metastasis and recurrence, TNBC complicates treatment options and poses significant challenges for clinical management, which ultimately leads to poor patient prognosis (3, 41). The absence of targeted therapies for TNBC underscores the urgent need for the creation of innovative treatment approaches to enhance patient outcomes. Recent advances in immunotherapy have opened new avenues for treating TNBC, emphasizing the importance of understanding the TME and the immune system’s role in tumor progression (34, 42). Given the pressing need for efficacious treatments, investigating the interactions of innovative therapies such as EA between immune and epigenetic modulation may provide valuable insights into bolstering anti-tumor immunity in TNBC patients.

This study investigates the potential of peritumoral EA to inhibit tumor growth in TNBC and its underlying immune mechanisms, specifically focusing on CD8+ T cell recruitment. By constructing TNBC orthotopic transplanted tumor in mice, we found that EA suppressed tumor growth, which has been proved in previous studies (23–25). Furthermore, our study demonstrates that peritumoral EA significantly increased intratumoral CD8+ T cell infiltration. The increase in CD8+ T cell infiltration following EA intervention suggests that this approach may activate and mobilize immune cells effectively, contributing to a more robust anti-tumor response. Notably, serum level of IL-6, TNFα, and TGFβ remained unchanged. The EA treatment didn’t lead to systemic inflammation, suggesting that it can improve the immune function in tumors without causing adverse effects, which is essential for developing effective cancer therapies. The study provides compelling evidence that peritumoral EA can significantly enhance the local immune response against TNBC. Meanwhile, EA remarkably increased tumoral CCL5, a chemokine essential for CD8+ T cell trafficking. It has been proved that CCL5 reconfigures the TME to favor T cell infiltration in pancreatic cancer (43). Peritumoral EA to localize immune activation offers a safer alternative to systemic checkpoint inhibitors. The spatial specificity may circumvent pitfalls of cytokine storm toxicity observed in existing immunotherapies. Future studies should explore whether EA can synergize with adoptive T cell therapies to enhance homing efficiency.

Epigenetic modifications play a critical role in the initiation and progression of various cancers (44, 45), including TNBC (46). Specifically, previous studies indicate that the down-regulation of HDAC1, an important epigenetic regulatory factor, is associated with enhanced immune responses, particularly in the recruitment of CD8+ T cells within the TME (11, 39, 47). Our bioinformatics analyses utilizing online open-source datasets demonstrated that the HDAC1 overexpression in TNBC correlates with poor prognosis, highlighting its significance in cancer progression. Moreover, histone modification, particularly the status of acetylation, significantly influences the expression of immune-related genes and modulates the tumor’s immune landscape (48). Recent single-cell RNA sequencing studies have revealed that HDAC1 overexpression in human BRCA is linked to exhausted CD8+ T cell phenotypes and reduced chemokine signaling (49). This suggests that the down-regulation of HDAC1 may represent a key pathway through which epigenetic modifiers enhance immune responses, particularly the recruitment of CD8+ T cells within the TME. Studies have confirmed that HDAC1 inhibition reinstated CCL5-driven CD8+ T cell recruitment in cancer (12, 14, 40). Mechanistically, it is proposed that EA derepresses CCL5 transcription by reducing HDAC1 binding, thereby facilitating CD8+ T cell recruitment. The results align with previous studies which highlighted the role of HDAC inhibition in promoting tumor immunity (12, 13, 15), which suggests that integrating epigenetic modifiers with complementary therapies could enhance treatment efficacy in various malignancies. The interplay between epigenetic regulation and immune modulation underscores the potential of targeted interventions in altering the TME, offering new avenues for therapeutic strategies against TNBC.

However, TNBC exhibits distinct epigenetic vulnerabilities (50). Unraveling the epigenetic vulnerabilities within TNBC holds immense promise for the exploration of innovative therapeutic strategies in TNBC management. Our finding revealed that the monotherapy of HDAC1 inhibitor enhanced CD8+ T cells of blood and spleen, while failed to maximize intratumoral CD8+ T cells, which supports this notion. Meanwhile, the monotherapy of HDAC1 inhibitor such as romidepsin elevated serum CCL5 rather than intratumoral CCL5, in which the systemic administration increased serum CCL5 could potentially activate metastasis-prone CCR5+ tumor cells (51). On the contrary, EA circumvented this risk by localized CCL5 production to the TME, not only avoiding off-target effects but also highlighting its precision as a localized epigenetic modulator. The spatial disconnect implies that systemic HDAC inhibition may inadvertently promote metastasis via upregulation of circulating CCL5, underscoring the need for localized modulation strategies like peritumoral EA. Therefore, the peritumoral EA’s spatial specificity resolves a key limitation of HDAC inhibitors. Despite concerns that HDAC1 inhibitor exacerbate systematic inflammation (14), EA counteracted these effects, maintaining cytokine homeostasis.

The limitations of this study warrant careful consideration. Primarily, the findings are based on preclinical murine models, which may not fully replicate the complexities of human TNBC. The absence of clinical validation restricts the applicability of the results to patient populations. Furthermore, while the study emphasizes the role of immune and HDAC1 modulation, it does not explore the long-term effects of such interventions, nor does it address the optimal treatment parameters for clinical settings. These factors underscore the need for further research, including clinical trials, to ascertain the translational potential of these findings.

## 5 Conclusion

This study provides compelling evidence that peritumoral EA significantly inhibits tumor growth in TNBC, primarily enhanced recruitment of CD8+ T cells and downregulated expression of HDAC1. The observed interplay between immune and epigenetic modulation for peritumoral EA presents a novel therapeutic strategy that could improve outcomes for patients with the aggressive cancer subtype of BRCA. Despite the limitations inherent in preclinical research, the findings lay a crucial foundation for future investigations aimed at translating the findings into clinical practice. The integration of non-invasive therapies such as EA with conventional treatments holds promise for improving cancer immunotherapy and clinical outcomes for patient with TNBC.

## Data Availability

The original contributions presented in the study are included in the article/**Supplementary Material**. Further inquiries can be directed to the corresponding authors.
