# Supplementary figures and images for: Electroacupuncture combined with HDAC1 inhibitor suppress tumor growth via improving the recruitment of intratumor CD8+ T cells for triple-negative breast cancer in mice
Source: Front Oncol. 2025 May 22;15:1584722. doi: 10.3389/fonc.2025.1584722 (PMC12137248; doi:10.3389/fonc.2025.1584722)

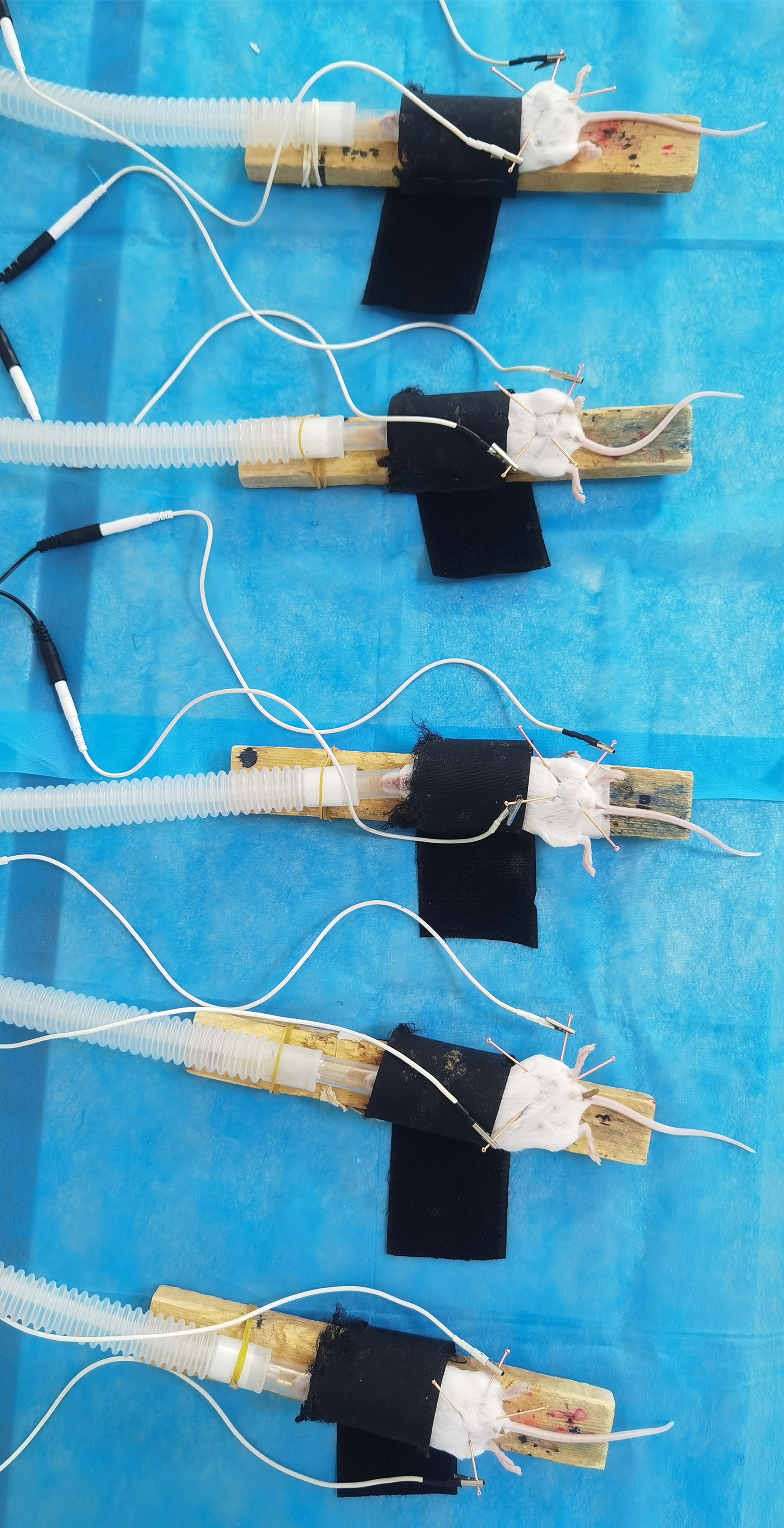

Supplement: Supplementary file 1 [file Image1.tif]

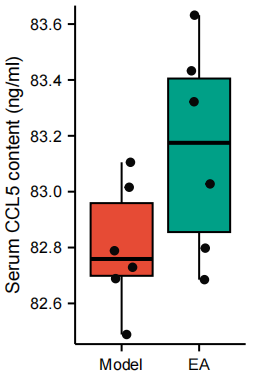

Supplement: Supplementary file 2 [file Image2.tif]

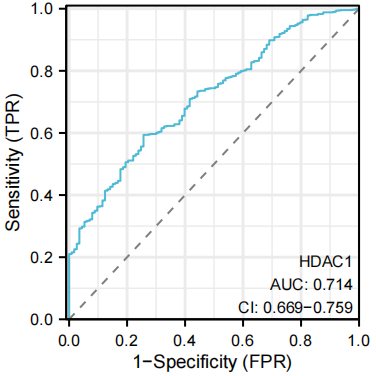

Supplement: Supplementary file 3 [file Image3.tif]

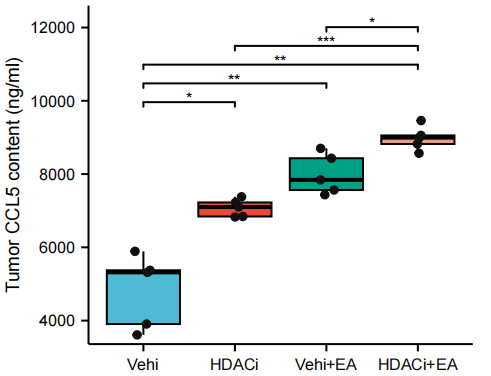

Supplement: Supplementary file 4 [file Image4.tif]
